# A Measure of Nutrition Security Using the National Health and Nutrition Examination Survey Dataset

**DOI:** 10.1001/jamanetworkopen.2024.62130

**Published:** 2025-02-28

**Authors:** Vibha Bhargava, Jung Sun Lee, Travis A. Smith, Sanchita Chakrovorty

**Affiliations:** 1 Department of Nutritional Sciences, University of Georgia, Athens; 2 Department of Agricultural and Applied Economics, University of Georgia, Athens

## Abstract

**Importance:**

Accurate and practical measures of nutrition security are needed to assess and monitor its prevalence and to identify its associated risk factors in the US.

**Objective:**

To propose a nutrition security measure derived from combining self-assessed food security and diet quality indicators available in the National Health and Nutrition Examination Survey (NHANES) and to assess the prevalence and associated sociodemographic and health factors of nutrition security.

**Design, Setting, and Participants:**

This cross-sectional study used data from 6 cycles of NHANES, which collects data on general health status and behaviors, dietary intake, physiological measurements, and sociodemographic characteristics, conducted from 2007 to 2018. All participants were adults aged 20 years or older. Statistical analysis was performed between October 2023 and April 2024.

**Exposures:**

Sociodemographic and health characteristics, including age, sex, race and ethnicity, marital status, household size, presence of children in the household, educational level, poverty to income ratio (PIR), Supplemental Nutrition Assistance Program (SNAP) participation, weight status, chronic conditions, and health insurance coverage.

**Main Outcomes and Measures:**

A nutrition security measure was derived from combining self-assessed food security, measured using the US Department of Agriculture Household Food Security Survey Module, and self-rated diet quality indicators. Four nutrition security status categories were created from dichotomized food security and diet quality measures: food secure with high diet quality (FSHD), food secure with low diet quality (FSLD), food insecure with high diet quality (FIHD), and food insecure with low diet quality (FILD). Only respondents classified as FSHD were considered to be nutrition secure.

**Results:**

The unweighted analytic sample included 28 898 NHANES participants. The weighted mean [SD] age of participants was 47.3 [14.5] years; 51.9% (weighted) of the sample was female; 11.1% identified as Black, 13.6% as Hispanic, and 67.4% as White individuals; and 35.6% of those surveyed were classified by the proposed measure as nutrition insecure (ie, FSLD, FIHD, or FILD). Of these participants, 20.2% (95% CI, 19.4%-21.0%) were categorized as being nutrition insecure due to FSLD, 8.4% (95% CI, 7.8%-9.1%) due to FIHD, and 7.0% (95% CI, 6.4%-7.6%) due to FILD. The remaining 64.4% (95% CI, 63.2%-65.7%) were classified as FSHD (ie, nutrition secure). Younger age (20-44 years: average marginal effect [AME], −0.193; 95% CI, −0.217 to −0.168), low income (PIR <1.30: AME, −0.111; 95% CI, −0.136 to –0.085), lower educational level (≤high school diploma: AME, −0.135; 95% CI, −0.156 to −0.114), racial and ethnic minority status (Hispanic: AME, −0.054; 95% CI, −0.075 to −0.032), SNAP participation (AME, −0.073; 95% CI, −0.099 to −0.047), obesity (AME, −0.118; 95% CI, −0.138 to −0.097), and self-reported fair or poor general health (AME, −0.239; 95% CI, −0.260 to −0.217) were factors associated with nutrition insecurity.

**Conclusions and Relevance:**

This cross-sectional study proposed a feasible and practical measure for assessing and monitoring nutrition security using validated measures in the NHANES. This study laid the groundwork for exploring other national datasets and performing regular data collection of key dimensions for nutrition security assessment and monitoring in the US.

## Key Points

**Question:**

How can an existing national dataset be used to measure nutrition security?

**Findings:**

In this cross-sectional study involving 28 898 participants in the National Health and Nutrition Examination Survey (NHANES), self-assessed food security and diet quality indicators were combined to construct and operationalize a measure of nutrition security using existing NHANES variables. The proposed measure estimated that 36% of adult participants had nutrition insecurity between 2007 and 2018.

**Meaning:**

The findings suggest that the proposed measure laid the groundwork for exploring other national datasets and performing regular data collection of key dimensions for nutrition security assessment and monitoring in the US.

## Teaser

This cross-sectional study describes the development and operationalization of a proposed method for assessing and monitoring nutrition security in US adults using the National Health and Nutrition Examination Survey (NHANES) dataset.

## Introduction

Poor nutrition has detrimental and long-lasting developmental, economic, social, and medical implications for individuals, families, communities, and nations.^1,2,3,4,5^ Food insecurity, defined as ‘‘the limited or uncertain availability of nutritionally adequate and safe foods or limited or uncertain ability to acquire acceptable foods in socially acceptable ways,”^6^^(p1560)^ has been monitored in the US for the past 30 years using standardized operational definitions and measurements as part of the National Nutrition Monitoring System. Increasing disparities in food insecurity, diet quality, and their associated health outcomes have brought attention to the emerging concept of nutrition insecurity, especially in terms of the capability to monitor it and its potential causes.^7,8^ Consequently, the US Department of Agriculture (USDA) announced the new Actions on Nutrition Security initiative to modernize and strengthen the capacity of nutrition assistance programs, promote nutrition education, and monitor progress in achieving nutrition security while promoting health equity.^9,10^

Unlike food insecurity, which has a well-established metric and consensus definition, nutrition security is gaining quick recognition from federal agencies, academic researchers, clinicians, and other stakeholders.^11,12^ The USDA defines nutrition security as “having consistent access, availability, and affordability of foods and beverages that promote well-being, prevent disease, and, if needed, treat disease, particularly among racial and ethnic minority populations, lower income populations, and rural and remote populations including Tribal communities and Insular areas.”^9^ Various conceptual frameworks and measures of nutrition security have been proposed to operationalize this definition. However, there are inconsistencies in these frameworks. The USDA conceptualizes nutrition security as food security with an added emphasis on diet quality and equity.^12^ Seligman et al^13^ conceptualize nutrition security and food security as distinct constructs, with nutrition security mediating the link between food security and health. The Center for Nutrition & Health Impact’s Household Nutrition Security (HSN) screener, which is not guided by any definition of nutrition security, assesses a household’s ability to acquire foods that promote health.^14^ A 2-item Nutrition Security Screener developed by Tufts University, Kaiser Permanente, and the Los Angeles County Department of Public Health evaluates difficulties in accessing and eating healthy foods and barriers to eating healthy over the past 12 months.^12^ Currently, there is no consensus definition, conceptualization, or standardized measure for nutrition security.

Consistent with the USDA’s conceptualization of nutrition security, we conducted this research to propose a nutrition security measure derived by combining self-assessed food security and diet quality indicators available in the the National Health and Nutrition Examination Survey (NHANES). In this study, we used a single-item, self-rated diet quality measure to assess healthy diets, a key construct of nutrition security.^13^ The concept of diet quality is complex, involving factors dependent on individual beliefs, expectations, and needs such as taste, appearance, culture, and convenience. Various diet quality measures exist, each with unique criteria for construction and validation against health outcomes, to evaluate dietary patterns and adherence to dietary recommendations.^15,16,17^ Unlike a priori diet quality measures (eg, Healthy Eating Index), the present self-rated measure assesses how an individual’s diet aligns with their perception of a healthy diet and may account for factors in food choice and diet quality, including individual characteristics (eg, physiological; sociodemographic; and knowledge, attitudes, and behaviors) as well as broader social, cultural, and environmental contexts.^18,19^ Studies evaluating single-item, self-rated diet quality measures show that individuals with higher self-rated diet quality tend to have higher scores on established diet quality indices but indicate that individuals tend to overestimate their diet quality.^20,21,22,23,24^

In the proposed metric operationalizing nutrition security, food security captures food hardships due to limited money and other resources for obtaining food, while self-rated diet quality assesses perceptions about the quality of foods and beverages available, purchased, and consumed by individuals. This combined food security and diet quality metric for nutrition security may be used to estimate the prevalence and associated sociodemographic and health factors of nutrition security in the US. The findings of this exploratory study could foster ongoing efforts to develop nutrition security metrics that complement existing food security measures and to elucidate the nature and magnitude of nutrition security.

## Methods

### Data and Sample

NHANES is an ongoing cross-sectional survey conducted by the National Center for Health Statistics (NCHS).^25^ The survey collects data on general health status and behaviors, dietary intake, physiological measurements, and sociodemographic characteristics via in-home questionnaires and in-person physical examinations. It uses a complex, multistage probability sampling design to select survey participants representative of the civilian, noninstitutionalized US population.^26^ The sample for this study was restricted to adults aged 20 years or older who were surveyed between the 2007 and 2018 survey cycles. NHANES is approved by the NCHS Research Ethics Review Board, and its participants provide written informed consent. The University of Georgia Institutional Review Board deemed this cross-sectional study exempt from review and the informed consent requirement because publicly available, deidentified NHANES data were used. We followed the Strengthening the Reporting of Observational Studies in Epidemiology (STROBE) reporting guideline.

### Outcome

Nutrition security is operationalized by integrating food security and self-rated diet quality measures. Household food insecurity was assessed using the validated 18-item USDA Household Food Security Survey Module for households with children or the 10-item module for households without children, with a 12-month reference period.^27^ Based on the number of affirmative responses in these surveys, food security was categorized as follows: full (0), marginal (1-2), low (3-7), and very low (8-18). Food security was dichotomized into food secure (households with full and marginal food security) and food insecure (households with low and very low food security). NHANES respondents were asked to assess their diet quality with this question: In general, how healthy is your overall diet? Response options included excellent, very good, good, fair, and poor. Excellent, very good, and good responses were categorized as high diet quality, and fair and poor responses were categorized as low diet quality following the widely used categorization of the self-reported general health question.^28,29^

Food security and diet quality binary variables were combined into 4 mutually exclusive categories of nutrition security: food secure with high diet quality (FSHD), food secure with low diet quality (FSLD), food insecure with high diet quality (FIHD), and food insecure with low diet quality (FILD). Consistent with the USDA’s definition of nutrition security, individuals with either low diet quality (regardless of food security status) or food insecurity (regardless of diet quality status) were considered to be nutrition insecure. Therefore, only individuals classified as FSHD were considered to be nutrition secure.

### Covariates

The existing literature on individual and household factors associated with food insecurity^30,31,32,33^ and diet quality^7,8,34,35^ guided the selection of covariates. Sociodemographic factors included age (20-44, 45-64, or ≥65 years), sex (male or female), race and ethnicity (Hispanic, non-Hispanic Black [hereafter Black], non-Hispanic White [hereafter White], or other [other Hispanic or other race, including multiracial]), educational level (≤high school diploma, some college or associate’s degree, or ≥bachelor’s degree), marital status (married or living with a partner; never married; or separated, widowed, or divorced), presence of children in the household, household size, family income as poverty to income ratio (PIR; <1.30, 1.30-2.99, or ≥3.00), and Supplemental Nutrition Assistance Program (SNAP) participation in the previous 12 months. Body mass index (BMI; calculated as weight in kilograms divided by height in meters squared) was the measure for weight and categorized as underweight or normal weight (BMI <25), overweight (BMI 25-29.9), and obesity (BMI ≥30). Chronic conditions, including diabetes, hypertension, high cholesterol, and heart disease (presence of at least 1 of the following: congestive heart failure, coronary heart disease, angina or angina pectoris, myocardial infarction, and stroke), were identified from self-reported diagnosis from a physician or another health professional. Self-reported general health status was dichotomized as excellent, very good, and good or fair and poor. Health insurance coverage categories included private only, public only, both private and public, and uninsured. Race and ethnicity data were self-reported and included in the study to examine potential differences in nutrition security status among racial and ethnic groups.

### Analytic Sample

The analytic sample was restricted to 33 412 respondents with nonzero mobile examination center survey weights. Due to missing data, 4514 adults were excluded from the analyses, resulting in a final analytic sample of 28 898 adults (eFigure in Supplement 1). Excluded individuals were more likely to be older, be from racial and ethnic minority groups, have a lower educational level, have a PIR below 1.30, self-report poor health status, have more diet-related chronic conditions, and be uninsured (eTable in Supplement 1).

### Statistical Analysis

We used descriptive statistics to describe the characteristics of the overall study population and by nutrition security status. Differences in sociodemographic and health characteristics by nutrition security status were estimated using *F* tests for continuous variables and Pearson χ^2^ tests for categorical variables. We used logistic regression to calculate *P* values for linear trends in nutrition insecurity over the study period. We used multinomial logit analysis to identify individual and household characteristics associated with the probability of being in each of the 4 discrete nutrition security status categories: FSHD, FSLD, FIHD, and FILD. Results from multinomial logit analysis were reported as percentage point changes in the probability of each category, calculated using average marginal effect (AME). All analyses used the mobile examination center survey weights and accounted for the complex sampling design.

Two-sided *P* ≤ .05 was considered statistically significant. Statistical analyses were conducted between October 2023 and April 2024 using Stata, version 18 (StataCorp LLC).

## Results

The unweighted analytic sample comprised 28 898 NHANES participants. In the weighted sample, the mean (SD) age of participants was 47.3 (14.5) years; 51.9% were female, and 48.1% were male; and 11.1% identified as Black, 13.6% as Hispanic, 67.4% as White, and 8.0% as other race and ethnicity (Table 1). Of these participants, 21.9% had a PIR below 1.30. A majority of the participants had overweight (32.8%) or obesity (37.8%) status, whereas 31.8% had hypertension and 11.7% reported having diabetes.

**Table 1.** Sociodemographic and Health Characteristics of Participants in the 2007 to 2018 National Health and Nutrition Examination Survey^a^

Characteristic	Participants, No. (weighted %)					
	Total	Nutrition secure: FSHD	Nutrition insecure			*P* value^b^
			FSLD	FIHD	FILD	
Unweighted sample, No.	28 898	17 097	5849	3285	2667	NA
Age, y						
20-44	12 271 (45.6)	6451 (41.4)	2775 (51.0)	1601 (56.4)	1444 (61.5)	<.001
45-64	9920 (35.7)	5775 (36.7)	2075 (36.8)	1142 (33.0)	928 (31.7)	
≥65	6707 (18.6)	4871 (21.9)	999 (12.2)	542 (10.6)	295 (6.8)	
Sex						
Female	14 924 (51.9)	8756 (52.7)	2967 (48.0)	1717 (53.7)	1479 (54.3)	<.001
Male	13 974 (48.1)	8341 (47.3)	2877 (52.0)	1568 (46.3)	1188 (45.7)	
Race and ethnicity^c^						
Hispanic	6986 (13.6)	3216 (9.6)	1725 (16.9)	1009 (23.2)	1036 (29.5)	<.001
Non-Hispanic Black	6196 (11.1)	3234 (8.6)	1444 (13.5)	861 (18.3)	657 (18.0)	
Non-Hispanic White	12 146 (67.4)	8087 (73.2)	2170 (63.3)	1064 (49.5)	825 (46.9)	
Other^c^	3570 (8.0)	2560 (8.6)	510 (6.3)	351 (9.0)	149 (5.5)	
Marital status						
Married or living with partner	17 235 (63.6)	10 941 (67.8)	3276 (59.3)	1668 (51.8)	1350 (51.3)	<.001
Widowed, divorced, or separated	6417 (18.3)	3523 (16.7)	1296 (18.7)	878 (23.9)	720 (25.2)	
Never married	5246 (18.1)	2633 (15.6)	1277 (22.0)	739 (24.3)	597 (23.5)	
Child present in household	11 998 (39.3)	6244 (35.0)	2608 (42.5)	1700 (51.5)	1446 (54.0)	<.001
Household size, mean (SD)	3.0 (1.3)	2.9 (1.2)	3.1 (1.3)	3.5 (1.7)	3.5 (1.8)	<.001
Educational level						
≤High school diploma	13 368 (38.0)	6519 (30.9)	3044 (44.3)	2004 (55.5)	1801 (64.3)	<.001
Some college or associate’s degree	8661 (31.5)	5138 (30.8)	1806 (33.3)	980 (34.0)	737 (30.2)	
≥Bachelor’s degree	6869 (30.4)	5440 (38.3)	999 (22.3)	301 (10.5)	129 (5.5)	
Family income: PIR						
<1.30	9331 (21.9)	3856 (14.4)	1756 (20.1)	2006 (54.6)	1713 (56.8)	<.001
1.30-2.99	9117 (28.6)	5260 (26.4)	2034 (31.6)	1038 (34.1)	785 (33.6)	
≥3.00	10 450 (49.5)	7981 (59.1)	2059 (48.3)	241 (11.3)	169 (9.6)	
Received SNAP benefits in past 12 mo						
Yes	6315 (15.7)	2262 (8.8)	1233 (15.1)	1535 (44.6)	1285 (46.8)	<.001
No	22 583 (84.3)	14 835 (91.2)	4616 (84.9)	1750 (55.4)	1382 (53.2)	
Health insurance						
Private only	11 870 (52.0)	8033 (57.5)	2507 (54.1)	776 (27.7)	554 (25.3)	
Public only	7697 (20.1)	3972 (17.2)	1444 (18.2)	1255 (34.4)	1026 (34.7)	<.001
Private and public	3235 (10.6)	2461 (13.0)	527 (8.2)	154 (4.4)	93 (3.6)	
Uninsured	6096 (17.3)	2631 (12.3)	1371 (19.6)	1100 (33.5)	994 (36.4)	
Weight status^d^						
Underweight or normal weight	8327 (29.4)	5611 (32.8)	1190 (20.5)	978 (30.7)	548 (21.8)	<.001
Overweight	9414 (32.8)	5970 (35.2)	1697 (28.6)	1034 (30.1)	713 (26.0)	
Obesity	11 157 (37.8)	5516 (32.0)	2962 (50.9)	1273 (39.3)	1406 (52.3)	
Health status						
Excellent, very good, or good	22 016 (82.6)	14 707 (90.3)	3669 (71.1)	2386 (76.2)	1254 (53.3)	<.001
Fair or poor	6882 (17.4)	2390 (9.7)	2180 (28.9)	899 (23.8)	1413 (46.7)	
Chronic conditions^e^						
Hypertension	10 422 (31.8)	6043 (30.9)	2213 (33.7)	1166 (30.9)	1000 (35.0)	.002
High cholesterol	9754 (32.8)	5959 (33.9)	2009 (33.6)	956 (26.5)	830 (28.2)	<.001
Diabetes	4390 (11.7)	2409 (10.8)	982 (13.3)	522 (12.6)	477 (14.4)	<.001
Heart disease^f^	3118 (8.4)	1781 (8.1)	599 (7.9)	398 (9.7)	340 (11.3)	<.001

Abbreviations: FIHD, food insecure with high diet quality; FILD, food insecure with low diet quality; FSHD, food secure with high diet quality; FSLD, food secure with low diet quality; NA, not applicable; PIR, poverty to income ratio; SNAP, Supplemental Nutrition Assistance Program.

^a^ Individuals were classified as food secure or food insecure and categorized as having high or low diet quality. These 2 binary variables were combined to create 4 nutrition security status categories: FSHD, FSLD, FIHD, and FILD.

^b^ *F* tests were used to calculate continuous variables and Pearsons χ^2^ tests used for categorical variables to identify statistically significant differences between nutrition security status categories.

^c^ Race and ethnicity were self-reported. Other included other Hispanic or other race, including multiracial.

^d^ Ascertained with body mass index (calculated as weight in kilograms divided by height in meters squared) as follows: <25 as underweight or normal weight, 25-29.9 as overweight, and ≥30 as obesity.

^e^ Chronic conditions were identified by an affirmative response to the question, “Has the doctor or other health professional ever told you…?” Since chronic conditions are not mutually exclusive, the column percentages do not add to 100%.

^f^ Defined as the presence of at least 1 of the following conditions: congestive heart failure, coronary heart disease, angina or angina pectoris, myocardial infarction, and stroke.

Using the proposed measure, 35.6% of participants were classified as nutrition insecure (ie, FSLD, FIHD, or FILD) at some point between 2007 and 2018 (Table 2). Of these participants, 20.2% (95% CI, 19.4%-21.0%) were classified as being nutrition insecure due to FSLD, 8.4% (95% CI, 7.8%-9.1%) due to FIHD and 7.0% (95% CI, 6.4%-7.6%) due to FILD. The remaining 64.4% (95% CI, 63.2%-65.7%) were classified as FSHD (ie, nutrition secure). Trend analysis indicated a significant decrease in nutrition security from 66.7% (95% CI, 64.0%-69.2%) in the 2007 to 2008 survey cycle to 59.8% (95% CI, 56.8%-82.6%) in the 2017 to 2018 cycle (*P* < .001 for linear trend), primarily due to an increase in prevalence of FILD from 4.7% (95% CI, 4.0%-5.6%) to 8.8% (95% CI, 7.2%- 10.7%) (Figure).

**Table 2.** Prevalence of Nutrition Security Status Categories Among Participants in the 2007 to 2018 National Health and Nutrition Examination Survey^a^

Self-rated diet quality	Self-rated food security, % (95% CI)	
	Food secure: full or marginal	Food insecure: low or very low
High quality: excellent, very good, or good	FSHD: 64.4 (63.2-65.7)	FIHD: 8.4 (7.8-9.1)
Low quality: fair or poor	FSLD: 20.2 (19.4-21.0)	FILD: 7.0 (6.4-7.6)

Abbreviations: FIHD, food insecure with high diet quality; FILD, food insecure with low diet quality; FSHD, food secure with high diet quality; FSLD, food secure with low diet quality.

^a^ Based on responses to the 18-item Household Food Security Survey Module, individuals were classified as food secure (full or marginal food security) or food insecure (low or very low food security). Based on responses to a single-item, self-rated diet quality question, individuals were categorized as having high (excellent, very good, or good) or low (fair or poor) diet quality. These 2 binary variables were combined to create 4 nutrition security status categories: FSHD, FSLD, FIHD, and FILD.

**Figure.**
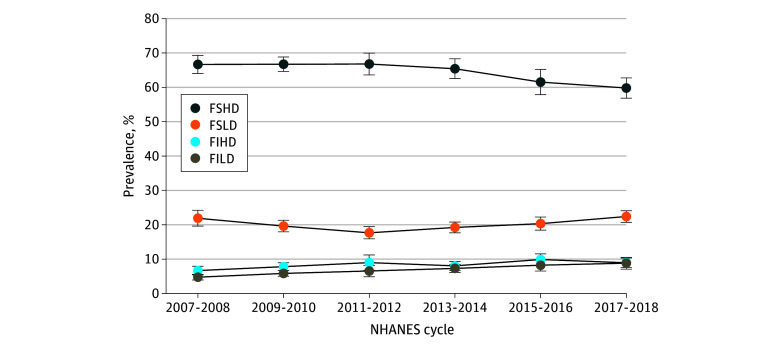
Nutrition Security Patterns Among US Adults in the 2007 to 2018 National Health and Nutrition Examination Survey (NHANES) by Survey Cycles Based on responses to the 18-item Household Food Security Survey Module, individuals were classified as food secure (full or marginal food security) or food insecure (low or very low food security). Based on responses to a single-item, self-rated diet quality question, individuals were categorized as having high (excellent, very good, or good) or low (fair or poor) diet quality. These 2 binary variables were combined to create 4 nutrition security status categories: food secure with high diet quality (FSHD), food secure with low diet quality (FSLD), food insecure with high diet quality (FIHD), and food insecure with low diet quality (FILD). Error bars represent 95% CIs.

Adults classified as FSLD as opposed to FILD were more likely to be White individuals (63.3% vs 46.9%), less likely to be of Hispanic ethnicity (16.9% vs 29.5%), and more likely to have at least a bachelor’s degree (22.3% vs 5.5%). No consistent patterns were observed in the prevalence of self-reported chronic conditions across the 4 nutrition security status categories.

Multivariate analysis showed that participants with younger age, lower educational level, and Hispanic ethnicity were more likely to be nutrition insecure (Table 3). Those aged 20 to 44 years (youngest group) had 19.3 percentage points (AME, −0.193; 95% CI, −0.217 to −0.168) lower probability of nutrition security than those aged 65 years or older (oldest group). Those with a high school diploma or less had 13.5 percentage points (AME, −0.135; 95% CI, −0.156 to −0.114) lower probability of being nutrition secure than those with a bachelor’s degree. Hispanic participants were 5.4 percentage points (AME, −0.054; 95% CI, −0.075 to −0.032) less likely to be nutrition secure than White participants. Additionally, males, nonmarried or nonpartnered adults, and those with children in the household were more likely to be nutrition insecure due to being classified as FSLD compared with other groups.

**Table 3.** Average Marginal Effects of Nutrition Security Status of Participants in the 2007 to 2018 National Health and Nutrition Examination Survey^a^

Characteristic	AME (95% CI)^b^			
	Nutrition secure: FSHD	Nutrition insecure		
		FSLD	FIHD	FILD
Age, y				
20-44	−0.193 (−0.217 to −0.168)	0.092 (0.071 to 0.114)	0.033 (0.020 to 0.045)	0.068 (0.057 to 0.079)
45-64	−0.132 (−0.156 to −0.108)	0.061 (0.041 to 0.082)	0.033 (0.022 to 0.044)	0.038 (0.031 to 0.044)
≥65	1 [Reference]	1 [Reference]	1 [Reference]	1 [Reference]
Sex				
Female	0.027 (0.013 to 0.041)	−0.026 (−0.038 to −0.015)	0.000 (−0.007 to 0.008)	−0.001 (−0.006 to 0.004)
Male	1 [Reference]	1 [Reference]	1 [Reference]	1 [Reference]
Race and ethnicity				
Hispanic	−0.054 (−0.075 to −0.032)	0.021 (0.005 to 0.037)	0.015 (0.002 to 0.029)	0.018 (0.008 to 0.027)
Non-Hispanic Black	−0.050 (−0.068 to −0.032)	0.027 (0.014 to 0.040)	0.014 (0.004 to 0.025)	0.009 (−0.001 to 0.018)
Non-Hispanic White	1 [Reference]	1 [Reference]	1 [Reference]	1 [Reference]
Other^c^	0.019 (−0.007 to 0.044)	−0.024 (−0.046 to −0.001)	0.016 (−0.000 to 0.033)	−0.011 (−0.025 to 0.002)
Marital status				
Widowed, divorced, or separated	−0.066 (−0.088 to −0.045)	0.035 (0.016 to 0.054)	0.012 (0.001 to 0.024)	0.019 (0.010 to 0.028)
Never married	−0.062 (−0.083 to −0.040)	0.057 (0.036 to 0.079)	0.002 (−0.009 to 0.014)	0.002 (−0.009 to 0.013)
Married or partnered	1 [Reference]	1 [Reference]	1 [Reference]	1 [Reference]
Child present in household				
Yes	−0.025 (−0.045 to −0.004)	0.024 (0.008 to 0.040)	−0.002 (−0.016 to 0.012)	0.003 (−0.007 to 0.013)
No	1 [Reference]	1 [Reference]	1 [Reference]	1 [Reference]
Household size	0.001 (−0.006 to 0.008)	−0.002 (−0.008 to 0.003)	0.003 (−0.002 to 0.008)	−0.002 (−0.005 to 0.001)
Educational level				
≤High school diploma	−0.135 (−0.156 to −0.114)	0.066 (0.046 to 0.085)	0.023 (0.009 to 0.036)	0.047 (0.038 to 0.055)
Some college or associate’s degree	−0.099 (−0.116 to −0.082)	0.039 (0.023 to 0.055)	0.026 (0.012 to 0.040)	0.034 (0.024 to 0.045)
≥Bachelor’s degree	1 [Reference]	1 [Reference]	1 [Reference]	1 [Reference]
PIR				
<1.30	−0.111 (−0.136 to −0.085)	−0.076 (−0.097 to −0.054)	0.112 (0.095 to 0.130)	0.074 (0.060 to 0.088)
1.30-2.99	−0.075 (−0.092 to −0.058)	−0.037 (−0.055 to −0.018)	0.066 (0.054 to 0.078)	0.047 (0.038 to 0.055)
≥3.00	1 [Reference]	1 [Reference]	1 [Reference]	1 [Reference]
Received SNAP benefits in past 12 mos				
Yes	−0.073 (−0.099 to −0.047)	−0.012 (−0.029 to 0.004)	0.053 (0.038 to 0.069)	0.032 (0.020 to 0.044)
No	1 [Reference]	1 [Reference]	1 [Reference]	1 [Reference]
Health insurance				
Public only	−0.009 (−0.027 to 0.008)	−0.024 (−0.040 to −0.007)	0.022 (0.011 to 0.034)	0.011 (0.001 to 0.021)
Private and public	0.004 (−0.030 to 0.037)	0.002 (−0.034 to 0.038)	−0.005 (−0.020 to 0.011)	−0.001 (−0.017 to 0.015)
Uninsured	−0.048 (−0.070 to −0.027)	0.001 (−0.019 to 0.022)	0.026 (0.014 to 0.039)	0.021 (0.011 to 0.030)
Private only	1 [Reference]	1 [Reference]	1 [Reference]	1 [Reference]
Weight status				
Overweight	−0.031 (−0.048 to −0.013)	0.034 (0.020 to 0.049)	−0.008 (−0.019 to 0.002)	0.005 (−0.004 to 0.013)
Obesity	−0.118 (−0.138 to −0.097)	0.106 (0.090 to 0.122)	−0.011 (−0.022 to −0.000)	0.023 (0.014 to 0.031)
Underweight or normal weight	1 [Reference]	1 [Reference]	1 [Reference]	1 [Reference]
Health status				
Fair or poor	−0.239 (−0.260 to −0.217)	0.185 (0.162 to 0.209)	−0.015 (−0.024 to −0.006)	0.068 (0.058 to 0.078)
Excellent, very good, or good	1 [Reference]	1 [Reference]	1 [Reference]	1 [Reference]
Chronic conditions^d^				
Hypertension	−0.013 (−0.028 to 0.002)	0.002 (−0.012 to 0.016)	0.004 (−0.005 to 0.012)	0.007 (−0.002 to 0.016)
High cholesterol	−0.012 (−0.028 to 0.003)	0.015 (−0.001 to 0.030)	−0.002 (−0.011 to 0.008)	−0.001 (−0.009 to 0.007)
Diabetes	0.004 (−0.016 to 0.024)	−0.011 (−0.029 to 0.006)	0.009 (−0.002 to 0.020)	−0.001 (−0.011 to 0.008)
Heart disease^e^	0.009 (−0.020 to 0.037)	−0.029 (−0.051 to −0.008)	0.010 (−0.003 to 0.024)	0.010 (−0.002 to 0.023)
Survey cycle				
2009-2010	0.000 (−0.029 to 0.028)	−0.019 (−0.043 to 0.005)	0.008 (−0.010 to 0.027)	0.011 (0.001 to 0.020)
2011-2012	0.002 (−0.023 to 0.028)	−0.029 (−0.054 to −0.004)	0.012 (−0.008 to 0.032)	0.014 (−0.000 to 0.029)
2013-2014	−0.004 (−0.032 to 0.024)	−0.020 (−0.044 to 0.004)	0.004 (−0.013 to 0.021)	0.020 (0.008 to 0.032)
2015-2016	−0.054 (−0.084 to −0.025)	−0.006 (−0.031 to 0.018)	0.027 (0.007 to 0.046)	0.034 (0.024 to 0.044)
2017-2018	−0.069 (−0.098 to −0.039)	0.009 (−0.016 to 0.033)	0.019 (0.001 to 0.037)	0.041 (0.026 to 0.056)
2007-2008	1 [Reference]	1 [Reference]	1 [Reference]	1 [Reference]

Abbreviations: AME, average marginal effect; FIHD, food insecure with high diet quality; FILD, food insecure with low diet quality; FSHD, food secure with high diet quality; FSLD, food secure with low diet quality; PIR, poverty to income ratio; SNAP, Supplemental Nutrition Assistance Program.

^a^ Based on responses to the 18-item Household Food Security Survey Module, individuals were classified as food secure (full or marginal food security) or food insecure (low or very low food security). Based on responses to a single-item, self-rated diet quality question, individuals were categorized as having high (excellent, very good, or good) or low (fair or poor) diet quality. These 2 binary variables were combined to create 4 nutrition security status categories: FSHD, FSLD, FIHD, and FILD.

^b^ Multinomial logistic regression model was used to estimate AMEs and 95% CIs for associations between sociodemographic and health characteristics and derived nutrition security status categories.

^c^ Race and ethnicity were self-reported. Other included other Hispanic or other race, including multiracial.

^d^ Self-reported chronic conditions were identified by an affirmative response to the question, “Has the doctor or other health professional ever told you…?”

^e^ Defined as the presence of at least 1 of the following conditions: congestive heart failure, coronary heart disease, angina or angina pectoris, myocardial infarction, and stroke.

The probability of being nutrition insecure due to FSLD was 7.6 percentage points (AME, −0.076; 95% CI, −0.097 to –0.054) lower for those with PIR below 1.30 compared with those with PIR at or above 3.00. Those who received SNAP benefits in the past 12 months were less likely to be nutrition secure (AME, −0.073; 95% CI, −0.099 to −0.047) and more likely to be nutrition insecure due to FIHD (AME, 0.053; 95% CI, 0.038-0.069) or FILD (AME, 0.032; 95% CI, 0.020-0.044). Uninsured adults were also less likely to be nutrition secure than those with private insurance (AME, −0.048; 95% CI, −0.070 to −0.027). Adults with obesity were less likely to be nutrition secure (AME, −0.118; 95% CI, −0.138 to −0.097) and more likely to be classified as FSLD (AME, 0.106; 95% CI, 0.090-0.122) or FILD (AME, 0.023; 95% CI, 0.014-0.031) compared with those with underweight or normal weight. Compared with adults reporting excellent, very good, and good general health status, those reporting fair and poor health had a lower probability of being nutrition secure (AME, −0.239; 95% CI, −0.260 to −0.217) and a higher probability of being classified as FSLD (AME, 0.185; 95% CI, 0.162-0.209) or FILD (AME, 0.068; 95% CI, 0.058-0.078). Presence of chronic conditions was not associated with nutrition security, with one exception. Adults with heart disease were less likely to be classified as FSLD (AME, −0.029; 95% CI, −0.051 to −0.008).

## Discussion

To address ongoing nutrition-related health disparities, it is critical to measure and monitor nutrition insecurity using valid and practical measures at the population level. This study operationalized nutrition security by combining answers to a food security questionnaire and a single-item self-assessment of diet quality within a nationally representative dataset. This derived measure was used to estimate the prevalence of nutrition insecurity and its association with individual characteristics. Based on this measure, between 2007 and 2018, 35.6% of NHANES participants aged 20 years or older had nutrition insecurity because they reported food insecurity, low diet quality, or both. Two recent studies using the HSN measure reported prevalence estimates ranging from 30% in a convenience sample of food-insecure households without children^36^ to 59% in a national sample of 1454 SNAP participants.^37^ The Nutrition Security Screener found that 36% of households with food security were nutrition insecure.^12^ The nutrition insecurity prevalence from this study cannot be directly compared with prevalence based on alternative measures and/or study populations.

In the proposed metric operationalizing the USDA’s nutrition security definition, individuals are considered to be nutrition insecure if they do not consume a nutritionally adequate diet, regardless of their economic access to adequate amounts of food. Available nutrition security measures cannot identify this FSLD group. For example, the HSN measure was based on the experiences of individuals with food insecurity in accessing a healthy diet.^14^ This study found that 20.2% of adults with food security were nutrition insecure due to poor self-rated diet quality (FSLD), a group that has received minimal attention in policy discussions. Although not experiencing food insecurity, those classified as FSLD may still face substantial health risks from poor diets. While research and policy discussions have focused on poor diet quality among individuals with food insecurity, understanding the facilitators and barriers to a healthy diet among individuals with food security is equally critical.

Nutrition insecurity due to food insecurity and high diet quality was more prevalent among Black and Hispanic participants. Tucker et al^37^ also observed higher odds of simultaneous occurrence of food insecurity and nutrition security among Hispanic compared with White individuals, suggesting potential limitations in the validity of the HSN measure for this population. Further research should examine racial and ethnic differences in the interpretation of healthy diet and food insecurity in general and in the context of the questions used in the present study.^18,38^

While factors associated with food insecurity and/or diet quality are also associated with nutrition insecurity, additional factors not examined in this study could affect nutrition security. The coexistence of food security and low diet quality, as well as food insecurity and high diet quality, may indicate more nuance in factors affecting dietary choices, such as the time and monetary cost of planning, preparing, and consuming healthy foods; the food environment; and personal tastes or preferences.^39^ These findings underscore the diverse underlying factors of nutrition security, suggesting that tailored strategies may be needed to address nutrition insecurity in the US. For example, federal food assistance programs, such as SNAP, focus on the financial barriers to purchasing food and may be limited in addressing poor dietary quality. SNAP-Ed, the nutrition education arm of SNAP, could play a critical role in addressing nonfinancial factors in nutrition insecurity through nutrition education and policy, systems, and environmental change interventions.^39,40^

This study demonstrated the feasibility of using measures from an existing nationally representative dataset to derive a nutrition security measure. A draft of the 2024 Farm Bill by the House of Representatives mandates the USDA to report annually on food security and diet quality in the US and to evaluate the implications of any policies for food security and diet quality.^41^ The operationalization of nutrition insecurity presents a practical metric for assessing the prevalence of nutrition insecurity at the national level using NHANES, which provides data on key dimensions of nutrition security, including diet, nutrition, food security, and health outcomes. However, NHANES lacks data on barriers to accessing healthful foods (eg, the local food environment). The USDA’s Food Acquisition and Purchase Survey (FoodAPS) conducted in 2012 to 2013 provides detailed household food environment data alongside food insecurity and diet quality measures. While useful for constructing the proposed nutrition security measure, FoodAPS has limited health information, restricting the ability to examine the association between the proposed measure and health outcomes. To better understand the nature, magnitude, and programmatic solutions of nutrition insecurity, it may be critical to link high-quality data across platforms, such as food assistance administrative data and health care use.

### Limitations

The proposed nutrition security measure has several limitations. First, this study’s prevalence estimate of nutrition security was sensitive to the bifurcation of food security and self-rated diet quality. Additionally, the prevalence estimates of the proposed measure are likely to be conservative if individuals overestimate their diet quality. As the definition and conceptualization of nutrition security evolves, future research should explore the implications of alternative categorizations for calculating prevalence estimates and identifying associations with characteristics. Second, while the single-item, self-rated diet quality measure has been used in prior research to assess diet quality perceptions and appears in several validated, large-scale, nationally representative surveys, further studies are needed to evaluate the diet quality measure’s validity and reliability against other validated measures and health outcomes. Third, due to data limitations, the proposed measure does not fully capture several key dimensions of nutrition security, including access, availability, and affordability.

## Conclusions

In this cross-sectional study, a feasible and practical measure was proposed for assessing and monitoring nutrition security using validated measures in the NHANES. The proposed measure estimated that 35.6% of US adults had nutrition insecurity between the 2007 and 2018 NHANES cycles. While rigorous development and validation studies, such as those conducted for the food insecurity measure, are needed for refining an accurate and practical measure of nutrition security, this study sheds light on the feasibility of leveraging existing nationally representative data to derive a nutrition security measure. It also laid the groundwork for exploring other national datasets for this purpose and performing regular data collection of key dimensions for nutrition security assessment and monitoring in the US.

## Notes

Supplement 1. **eFigure.** Sample Selection Flowchart **eTable.** Sample Characteristics of Included and Excluded Participants, National Health and Nutrition Examination Survey 2007-2018

Supplement 2.
Data Sharing Statement
