# Supplementary material for: A Measure of Nutrition Security Using the National Health and Nutrition Examination Survey Dataset
Source: JAMA Netw Open. 2025 Feb 28;8(2):e2462130. doi: 10.1001/jamanetworkopen.2024.62130 (PMC11871541; doi:10.1001/jamanetworkopen.2024.62130)
Supplement: Supplement 1. — eFigure. Sample Selection Flowchart eTable. Sample Characteristics of Included and Excluded Participants, National Health and Nutrition Examination Survey 2007-2018 [file jamanetwopen-e2462130-s001.pdf]

Supplemental Online Content

Bhargava V, Lee JS, Smith TA, Chakrovorty S. A measure of nutrition security using the National Health and Nutrition Examination Survey dataset. *JAMA Netw Open*. 2025;8(2):e2462130. doi:10.1001/jamanetworkopen.2024.62130

eFigure. Sample Selection Flowchart

eTable. Sample Characteristics of Included and Excluded Participants, National Health and Nutrition Examination Survey 2007-2018

This supplemental material has been provided by the authors to give readers additional information about their work.

© 2025 Bhargava V et al. *JAMA Network Open*

eFigure. Sample Selection Flowchart

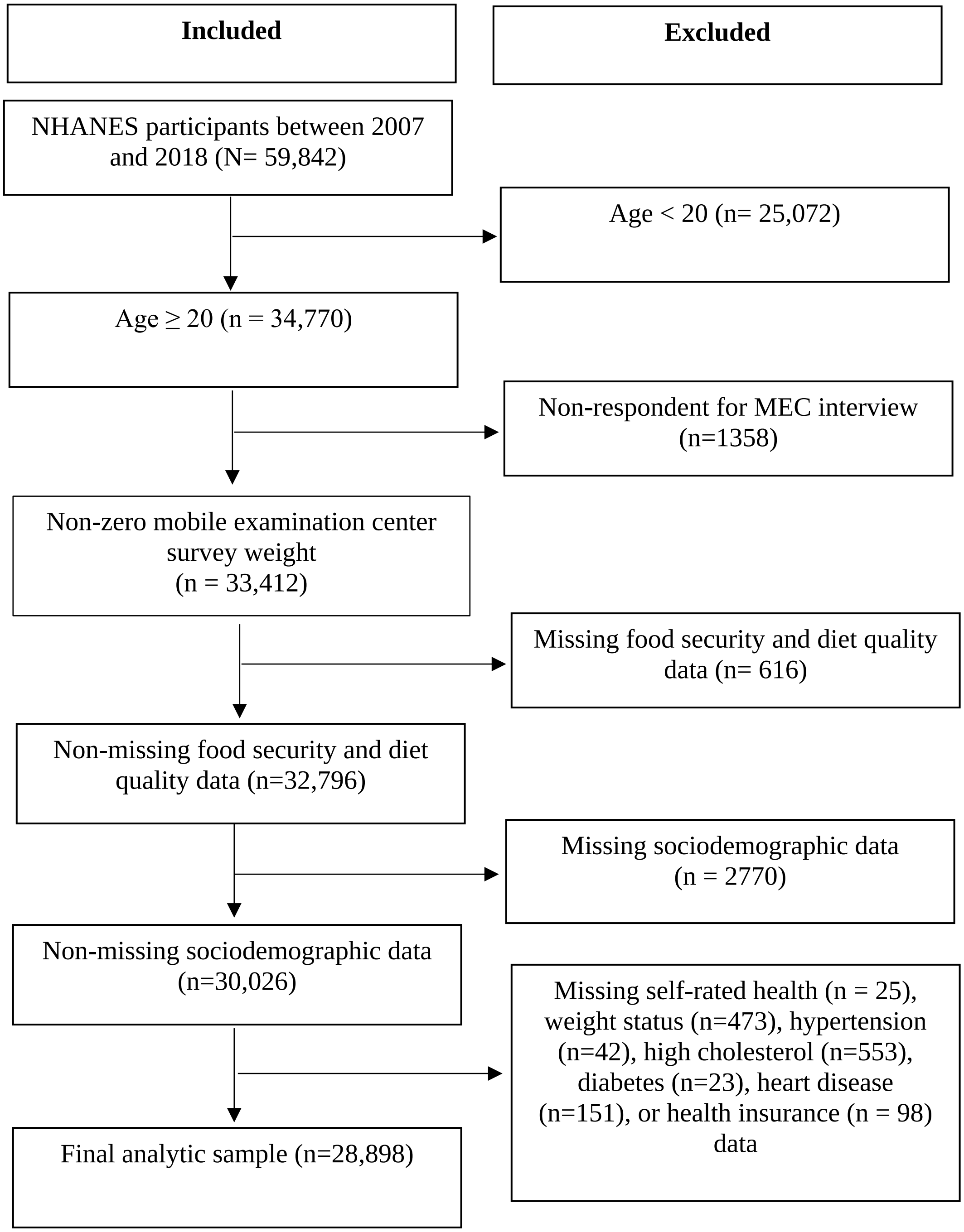

Abbreviations: NHANES, National Health and Nutrition Examination Survey

© 2025 Bhargava V et al. *JAMA Network Open*

eTable. Sample Characteristics of Included and Excluded Participants, National Health and Nutrition Examination Survey 2007-2018

	Participants No. (weighted %)		
	Study Participants (N= 28898)	Excluded Participants (N=4514)	P value[a]
**Age (Years)**			
20-44	12271 (45.61)	1654 (42.58)	<0.001
45-64	9920 (35.75)	1514 (33.3)	
>=65	6707 (18.64)	1346 (24.12)	
**Gender**			
Male	13974 (48.07)	2206 (48.36)	0.769
Female	14924 (51.93)	2308 (51.64)	
**Race and Ethnicity**			
Non-Hispanic White	12146 (67.35)	1378 (53.79)	<0.001
Non-Hispanic Black	6196 (11.05)	1036 (14.25)	
Hispanic	6986 (13.63)	1519 (21.7)	
Other [b]	3570 (7.96)	581 (10.26)	
**Marital Status**			
Married or living with partner	17235 (63.56)	2457 (57.05)	<0.001
Widowed, divorced, or Separated	6417 (18.29)	1109 (20.94)	
Never married	5246 (18.15)	927 (22.02)	
Child present in household	11998 (39.25)	1564 (32.41)	<0.001
Household size, mean(SD)	3.02 (1.31)	3.30 (1.66)	<0.001
**Education**			
<=High school diploma	13368 (38.04)	2559 (48.37)	<0.001
Some college or associate's degree	8661 (31.52)	1127 (28.82)	
Bachelor's degree	6869 (30.44)	784 (22.8)	
**Family income: PIR**			
<1.30	9331 (21.92)	525 (32.23)	<0.001
1.30-2.99	9117 (28.61)	446 (34.9)	
>=3.00	10450 (49.48)	278 (32.87)	
**Received SNAP benefits in the past 12 months**			
Yes	6315 (15.73)	842 (17.29)	0.087
No	22583 (84.27)	2966 (82.71)	
**Health**			
Weight status[c]			

© 2025 Bhargava V et al. *JAMA Network Open*

Underweight or Normal weight	8327 (29.38)	1151 (31.11)	0.111
Overweight	9414 (32.78)	1398 (33.45)	
Obesity	11157 (37.84)	1492 (35.44)	
**Health status**			
Excellent, very good, good	22016 (82.6)	3030 (74.24)	<0.001
Fair or poor	6882 (17.4)	1459 (25.76)	
**Chronic conditions[d]**			
Hypertension	10422 (31.76)	1759 (34.94)	0.002
High Cholesterol	9754 (32.8)	1354 (32.39)	0.669
Diabetes	4390 (11.72)	859 (14.58)	<0.001
Heart disease[e]	3118 (8.43)	576 (11.25)	<0.001
**Health insurance**			
Private only	11870 (52.04)	1312 (37.74)	<0.001
Public only	7697 (20.1)	1479 (26.83)	
Private and Public	3235 (10.61)	505 (12.33)	
Uninsured	6096 (17.25)	1120 (23.1)	

[a] *F* -tests used to calculate for continuous variables and Pearsons $\chi 2$ tests used for categorical variables to determine identify statistically significant differences between the included and excluded sample.
[b] Race and /ethnicity were self-reported. Other category included other Hispanic or other race, including multiracial.
[c]Weight was ascertained with body mass index (BMI; calculated as weight in kilograms divided by height in meters squared). BMI was categorized into the following groups: <25 as underweight or normal weight (BMI<25), 25-29.9 as overweight (BMI 25–29.9), and ≥30 as obesity. (BMI≥30)
[d] Chronic conditions were identified by an affirmative response to the question, "Has the doctor or other health professional ever told you…?" Since the chronic conditions are not mutually exclusive, the column percentages do not add to 100%.
[e] Defined as the presence of at least one of the following conditions: congestive heart failure, coronary heart disease, angina or angina pectoris, myocardial infarction, and stroke.

© 2025 Bhargava V et al. *JAMA Network Open*
