# Supplementary material for: A Measure of Nutrition Security Using the National Health and Nutrition Examination Survey Dataset
Source: JAMA Netw Open. 2025 Feb 28;8(2):e2462130. doi: 10.1001/jamanetworkopen.2024.62130 (PMC11871541; doi:10.1001/jamanetworkopen.2024.62130)
Supplement: Supplement 2. — Data Sharing Statement [file jamanetwopen-e2462130-s002.pdf]

Data Sharing Statement

Bhargava. A Measure of Nutrition Security Using the National Health and Nutrition Examination Survey Dataset. *JAMA Netw Open*. Published February 28, 2025.
doi:10.1001/jamanetworkopen.2024.62130

Data
Data available: No

Additional Information
Explanation for why data not available: The data (National Health and Nutrition Examination Survey) used in this study are available publicly via the Centers for Disease Control and Prevention website.
